# Establishment of hospital-based surgical residency programs as a health policy for surgical needs provision: an Indonesian perspective

**DOI:** 10.1097/MS9.0000000000001130

**Published:** 2023-08-03

**Authors:** Kiki Lukman

**Affiliations:** Department of Surgery, Faculty of Medicine, Universitas Padjadjaran, Bandung, Indonesia

**Keywords:** education, hospital-based residency, residency, surgical education, university-based residency

## Abstract

**Objective:**

One of the health interventions that can be implemented cost-effectively in developing countries is the provision of basic surgical procedures. One way to increase access to surgical services is to increase the production of surgeons, in this case through increasing the surgical residency program. There are similarities between university-based surgical residency programs and hospital-based surgical residency programs. However, in Indonesia, the differences between them are stark; the establishment of hospital-based surgical residency programs is challenging and requires collaboration between the government, university, college of surgery, and private sector.

**Design:**

The author reviewed the literature on post-graduate medical education for surgical programs and the contrast between university-based and hospital-based surgical residency programs.

**Setting:**

Information collected from the Indonesian Health Education Independent Accreditation Institution and varied literature was included.

**Participants:**

Not applicable.

**Result:**

In Indonesia, the implementation of an academic surgery environment in the hospital-based surgical residency program is challenging, and the implementation of the academic health system can increase student intake and provide more both from the university and the hospital-based surgical residency program.

## Introduction

Highlights

- Basic surgical needs are still scarce in developing countries.
- The production of surgeons is one of the keys to overcoming the provision of surgical needs.
- Hospital-based surgical residency is lacking in an academic surgery environment.
- Hospital-based surgical residency in Indonesia needs to be supported by the university.

‘We need to establish hospital-based residency programs as the newest system for increasing the number and distribution of specialist doctors’, says the statement of Indonesia’s Ministry of Health.

The difference in the ratio of medical doctors per 10 000 people in developed countries and developing countries is tremendous. World Health Organization (WHO) data in 2020 showed that in the United States of America, the ratio was 35.55, in the United Kingdom the ratio was 31.71, and in Canada, the ratio was 24.64. Meanwhile, in developing countries such as India, the ratio was 7.27, and in Indonesia, it was 6.95^1^. When viewed from the point of view of surgical needs, more than half of the world’s population lacks access to basic surgical procedures^2–4^.

There are three delays in surgical services in developing countries, including Indonesia. First, delay in seeking care is influenced by community awareness, culture, and beliefs. Second, delay in reaching care depended on geographical factors, long distances, and poor transportation. Third, delays in receiving care are affected by infrastructure and hospital resources. Indonesia, as one of the four most populated countries with a population of over 270 million people, spreads across Indonesia’s archipelago, which consists of more than 16 thousand islands, making the three delays in surgical services worse^2,3,5^.

One of the health interventions that can be implemented cost-effectively in developing countries is the provision of basic surgical procedures. As many as five billion people still have difficulty getting services for basic surgical needs such as appendicitis or surgical procedures for broken bones. Basic surgical procedures, as the name already stated, are really basic, relatively easy to perform, and relatively cheap procedures, but their differences make it possible to save someone’s life. Essential surgery ranks as many as 44 basic surgical procedures that are very essential to implement and can be accessed equally in developing countries. The number of deaths that can be prevented by the distribution of basic surgical procedures reaches 1.5 million people each year, or 6–7% of preventable deaths in developing countries^6–9^. One way to increase access to surgical services is to increase the production of surgeons, in this case, through the surgical residency program.

## Surgical training program in Indonesia

Indonesia’s independence was declared in 1945. The surgical training program before the 1960s was based on surgical apprenticeships in the government’s teaching hospital. The College of Surgeons of Indonesia (CSI) was established in 1967 to govern the administration, curriculum, finance, quality assurance of the training program, and national board examination for the surgical training program. Since the 1980s, the surgical training program has been governed and administered by the university in collaboration with the teaching hospital and CSI. Until now, university-based surgical residency programs (UBRP) have had many specialty and subspecialty training programs in surgery, and the number of surgeons who graduated from UBRP has not met the ratio of doctors to the population in Indonesia, especially in remote areas. Another rising issue is the high education cost of surgical training programs because the surgical resident and trainees should pay the tuition fees as university students, but the teaching hospital has no obligation to provide a salary for their contribution.

## University-based surgical residency programs versus hospital-based surgical residency programs

Currently, the majority of surgical residency programs are carried out by UBRP. This education system involves a university, a university hospital or tertiary general hospital, and a college of surgery. Whereas hospital-based surgical residency programs (HBRP) or community-based surgical residency programs are non-university-based residency programs that involve the hospital and college of surgery^10–12^.

The university as an institution plays a role in the student selection process, basic lectures, clinical practicum, and the university also provides opportunities for students to be involved in research through research funding grants that can be submitted by lecturers, providing opportunities for cross-sectoral or other university research within and abroad, and community service activities. The university also provides opportunities for cooperation with other hospitals as a network of practice locations for surgical residents because, at the main hospital, the cases that come are cases with a high degree of difficulty, needing more medical supervision, and chronic illnesses, and for surgical residents, more common cases and basic procedures are prominent in the district or smaller hospitals on a daily basis.

The tertiary general hospital or university hospital is a place for surgical residents to learn and practice surgical skills. The hospital also creates an environment for surgical residents to work together with other departments, such as anesthesiologists, internal medicine, radiologists, and others, to provide the best health services to patients. The College of Surgery as an organization provides guidance in the form of a surgical curriculum that must be followed by each study program, provides a basic or advanced training program for surgical residents, and acts as a national board of surgical examiners.

Ideally, the establishment of hospital-based surgical residency programs can accelerate the production of surgeons, which in the future is expected to make surgical services more equitable, especially in remote areas. The advantages of hospital-based surgical residency programs or community-based residency programs, although less obvious, are that their services can be delivered directly to the local community^12,13^. Community hospitals tend to be smaller but intimately connected to the local population. One of the highlighted advantages of hospital-based surgical residency is that the resident becomes the hospital worker, who gets a salary, and the tuition fees are relatively lower, which will have an impact on increasing interest in medical doctors becoming surgeons.

## The challenges for establishment of the hospital-based surgical residency programs

The challenge that must be faced in building a HBRP is to provide equal opportunities to surgical residents with the opportunities that can be achieved by taking a UBRP. The UBRP tends to receive more supervised surgical opportunities, scheduled training and teaching, and complex surgical cases, whereas the HBRP has more exposure to independent surgical experiences. Academic surgery environments also need to be established in the HBRP, including academic research, collaboration research, practical skill laboratories, and qualified teaching and learning facilities. Selecting and preparing a hospital that is suitable for a hospital-based surgical residency program must meet certain standards to be at least similar to a government’s tertiary general hospital, a government’s national referral hospital, or a private tertiary hospital.

## Key movements for increasing the production of surgeons

Data in 2023 shows that there are 92 university-based medical faculties in Indonesia, with 33 medical faculties that have been accredited, and there are 17 university-based surgical residency programs that have been accredited^14,15^. The implementation of the academic health system (AHS) by the UBRP allows the university to establish collaboration with more than one hospital, thus increasing its capabilities to increase student intake each year and provide more surgeons. The government, as the stakeholder, needs to give thoughtfulness to the health policy to provide scholarships for surgical residents and encourage the hospital to give surgical residents salaries based on their performance. The government’s collaboration with the private sector may possibly increase the number of university-based medical faculties to establish a specialty surgery program as a university-based surgical residency program.

Hospital-based surgical residency programs may be established with the collaboration of the UBRP and the College of Surgery. Initially, HBRP may be integrated into AHS with the surgical resident listed as a university student. In the future, the HBRP can act independently and receive surgical residents as their own students.

## Conclusion

The stakeholders and program directors need to ensure the quality of the education, surgical practice coverage, and exposure to a wide number of cases for both the UBRP and the NBRP residents to produce highly competent surgeons and meet the expectations of changing community surgical needs.

## Ethical approval

Ethics approval was not required for this perspective article.

## Consent

Informed consent was not required for this perspective article.

## Sources of funding

The author received no funding for this paper.

## Author contribution

Kiki Lukman participated in the conception and design of the study, the drafting, and the finalization of the manuscript.

## Conflicts of interest disclosure

None to be declared.

## Research registration unique identifying number (UIN)

Not applicable.

## Guarantor

Kiki Lukman acts as the author and the guarantor of this article. The author is fully responsible for the work, data accessibility, and decision to publish.

## Provenance and peer review

Not commissioned, externally peer-reviewed.

## Data availability statement

All data within the article are available upon request to the author.
